# Diagnóstico genético prenatal de enfermedades monogénicas

**DOI:** 10.1515/almed-2022-0086

**Published:** 2023-02-20

**Authors:** Carmen Prior-de Castro, Clara Gómez-González, Raquel Rodríguez-López, Hada C. Macher, en representación de la Comisión de Diagnóstico Prenatal y la Comisión de Genética de la Sociedad Española de Medicina de Laboratorio

**Affiliations:** Servicio de Genética, Hospital Universitario La Paz, Madrid, España; Laboratorio de Genética, Servicio Análisis Clínicos, Consorcio Hospital General Universitario, Valencia, España; Departamento de Bioquímica Clínica, Hospital Universitario Virgen del Rocío de Sevilla, Sevilla, España; Instituto de Investigaciones Biomédicas de Sevilla, IBIS, Universidad de Sevilla, Sevilla, España

**Keywords:** asesoramiento gen, diagnóstico genético prenatal, enfermedades monogénicas, recomendaciones, técnicas moleculares

## Abstract

El diagnóstico genético prenatal de enfermedades monogénicas es un proceso que engloba el conjunto de técnicas moleculares dirigidas a caracterizar molecularmente una posible enfermedad monogénica en el feto durante el embarazo. Actualmente, el diagnóstico genético prenatal puede realizarse de manera invasiva o no invasiva. Debemos diferenciar “NIPD” (del inglés: *non invasive prenatal diagnosis*) que se considera diagnóstico de “NIPT” (del inglés: *non invasive prenatal test*) que se considera prueba de cribado y tendrá que confirmarse por técnicas invasivas. Las diferentes metodologías disponibles y empleadas pueden ir dirigidas a la detección de variante/s genética/s causal/es previamente caracterizada/s en la familia, la detección del haplotipo de riesgo asociado a la mutación familiar y/o la búsqueda de posibles variante/s patogénicas en un gen asociado a una sospecha diagnóstica. Se revisarán los aspectos relevantes del diagnóstico genético prenatal de las enfermedades monogénicas. El objetivo es la exposición de las principales técnicas moleculares disponibles y empleadas en la actualidad, detallando sus indicaciones, limitaciones y recomendaciones analíticas, así como la normativa que regula el asesoramiento genético. La evolución permanente y exponencial en la aplicación asistencial de las técnicas genómicas, facilita progresivamente el acceso a una caracterización molecular exhaustiva, obligando a una constante actualización homogénea de los laboratorios.

## Introducción

El diagnóstico genético prenatal de enfermedades monogénicas es un proceso que engloba el conjunto de técnicas moleculares dirigidas al diagnóstico genético de una enfermedad monogénica en el feto durante el embarazo. Las enfermedades denominadas monogénicas son las relacionadas directamente con la alteración de un único gen, habiéndose aceptado su consideración como enfermedades raras por su baja prevalencia (afectan a menos de 5 por cada 10,000 habitantes) [1]. Las enfermedades monogénicas analizadas más frecuentemente mediante diagnóstico prenatal son: fibrosis quística, corea de Huntington, distrofia miotónica tipo 1, distrofia muscular de Duchenne, distrofia muscular fascioescapulohumeral, enfermedad de Gaucher, enfermedad de Pompe, ataxia de Friedreich, poliquistosis renal y neurofibromatosis, principalmente.

De acuerdo a la normativa vigente en España (Orden SSI/2065/2014 (BOE del 6 de noviembre) [2], el estudio genético prenatal se debe indicar cuando se cumplan los siguientes criterios:

– Sospecha de que el feto tenga un riesgo elevado de padecer una enfermedad genética grave y/o que sus progenitores presenten una historia familiar de una patología genética severa.
– La enfermedad sospechada se atribuya a una alteración genética conocida y pueda ser identificada mediante su análisis genético específico.
– De las conclusiones obtenidas de los resultados del estudio genético prenatal propuesto, puedan derivarse decisiones que contribuyan al manejo clínico más preciso de la gestación, del recién nacido o conducir a decisiones reproductivas.

Actualmente, el diagnóstico genético prenatal puede realizarse de manera invasiva (muestras procedentes de vellosidades coriales o amnios fetal) o no invasiva (la muestra de partida es la sangre periférica de la gestante, sin que pueda afectar físicamente a los tejidos de origen fetal) [3].

El ADN circulante fetal es una pequeña fracción del ADN que circula en el plasma materno (entre un 3 y un 20%). Está constituido por fragmentos cortos, en su mayoría menores de 150 pares de bases, y se postula que su origen es apoptótico proveniente de células fetales de la placenta. Los fragmentos de ADN fetal son detectables a partir de la sexta semana de gestación aumentando su proporción conforme avanza la gestación, y son indetectables en el plasma materno a las 48 horas tras el parto, siendo eliminados en su mayoría en las 2 primeras horas postparto [4], por lo que no se podría detectar ADN de un embarazo anterior. La fracción fetal se ve afectada por algunos factores como el peso de la gestante, hábitos tóxicos durante el embarazo, la existencia de un cáncer en la madre o una enfermedad autoinmune activa [5] así como si existe patología asociada a la placenta, como la preeclampsia [6], [7], [8], entre otros. Hay un importante número de factores que afectan la fracción fetal: determinadas características maternas, características placenta-feto, factores experimentales y métodos de cálculo [9].

Por tanto, es posible amplificar genes en el plasma de la gestante para detectar variantes genéticas asociadas a enfermedades monogénicas [10] que, si están ausentes en la madre, inequívocamente pertenecen al feto (origen paterno o *de novo*). En este caso, el análisis es dirigido y se considera diagnóstico, denominándose técnicas de “NIPD” (del inglés: *non invasive prenatal diagnosis*). Cuando se quiere diagnosticar en el feto una variante heredada de la madre, cada vez es más asequible el diagnóstico de enfermedades recesivas y/o ligadas al cromosoma X, así como las mutaciones dominantes heredadas de la madre, gracias a la secuenciación masiva y PCR digital. En este caso se considera prueba de cribado, denominándose técnicas de “NIPT” (del inglés: *non invasive prenatal test*) y tendrá que ser confirmada por técnicas invasivas.

Aunque el ADN fetal es detectable a partir de la sexta semana de gestación, la detección de variantes genéticas fetales en el plasma de la madre, mediante técnicas de biología molecular, se considera segura y diagnóstica en el NIPD, desde la décima semana de gestación y se puede tener información fiable en la que basar decisiones. Aunque en el NIPT deberá ser confirmado por técnicas invasivas.

## Objeto y campo de aplicación

La presente revisión tiene por objeto revisar los aspectos más relevantes del diagnóstico genético prenatal de las enfermedades monogénicas. Recopilando las principales técnicas moleculares que se aplican tanto en el diagnóstico invasivo como no invasivo, detallando sus indicaciones, limitaciones y recomendaciones analíticas. En un último apartado se incluyen las recomendaciones del asesoramiento genético imprescindible en el diagnóstico genético. Pretendemos que pueda ser utilizado como una fuente de consulta y ayude a enfocar e interpretar el diagnóstico genético de las enfermedades monogénicas.

## Indicaciones

El diagnóstico prenatal de enfermedades monogénicas se ofrece en los siguientes casos:

– Parejas con alto riesgo de transmitir una enfermedad monogénica al feto en los que previamente se ha caracterizado la/s alteración/es genética/s causal/es:
  – Uno de los progenitores presenta una alteración genética causante de una enfermedad monogénica autosómica dominante o ligada al X dominante.
  – La gestante sea portadora de una alteración genética causante de una enfermedad ligada al X recesiva.
    En estos casos se puede determinar el sexo fetal mediante técnicas no invasivas y en los fetos varones determinar el genotipo del feto mediante técnicas invasivas.
  – Ambos progenitores son portadores de una alteración genética causante de una enfermedad monogénica autosómica recesiva.

En determinados casos se contempla la donación de gametos o un diagnóstico genético preimplantacional (DGP, actualmente denominado PGT: *p* *re-implantation* *genetic testing*) para concebir una descendencia sana.

– Parejas en riesgo de transmitir al feto alteraciones genéticas no identificadas, causantes de una enfermedad monogénica, pero sí identificado el haplotipo de riesgo asociado a su desarrollo.
– Hijo previo diagnosticado de una enfermedad monogénica causada por una variante patogénica *de novo* y en la que existe riesgo de mosaicismo germinal.
– Presencia de hallazgos ecográficos en el feto, sugestivos de una enfermedad monogénica.

## Técnicas moleculares

En el abordaje molecular del diagnóstico prenatal se utilizan diferentes técnicas en función de la patología, el tipo de variante/s a estudio, su eficiencia y los tiempos de respuesta que permita.

Las diferentes metodologías disponibles y empleadas pueden ir dirigidas a la detección de la/s variante/s genética/s causal/es previamente caracterizada/s en la familia, a la detección del haplotipo de riesgo asociado a la mutación familiar o a la búsqueda de posibles variante/s patogénicas en un gen asociado a una determinada enfermedad sospechada.

La Tabla 1 detalla la utilidad de las principales técnicas empleadas en la actualidad en diagnóstico prenatal invasivo o no invasivo.

**Tabla 1:** Técnicas empleadas en función del tipo de diagnóstico prenatal.

**1. Diagnóstico genético prenatal invasivo.**

Secuenciación Sanger
MLPA
Array-CGH/Array de SNP
Estudio de microsatélites mediante PCR
TP-PCR
Amplificación específica de alelos (*ARMS*) (paneles de mutaciones más prevalentes de una enfermedad concreta)
NGS

**2. Diagnóstico genético prenatal no invasivo**

**2.1. Detección de alteraciones genéticas ausentes en la madre (NIPD)**
PCR a tiempo real con sondas *Taqman*
HRM y COLD-HRM
**2.2. Detección de alteraciones genéticas heredadas de la madre (NIPT), padre o *de novo*** ^***a***^
PCR digital
NGS

^a^En el caso de variantes patogénicas *de novo* detectadas en hijo previo con riesgo de mosaicismo germinal. MLPA, *multiplex ligation probe amplification*; TP-PCR, *triplet repeat primed* PCR; ARMS, *amplification-refractory mutation system*; NGS, *next generation sequencing*; HRM, *high resolution melting*.

### Secuenciación Sanger

La secuenciación por el método Sanger permite secuenciar una región del ADN previamente amplificada mediante la reacción en cadena de la polimerasa (PCR) [11]. Se emplea en el diagnóstico prenatal para la detección de mutaciones puntuales (cambio de sentido o missense, sin sentido o nonsense), pequeñas deleciones/duplicaciones o pequeñas inserciones. Es la técnica utilizada en el estudio de variantes patogénicas familiares en el feto en el diagnóstico prenatal invasivo. La baja concentración de ADN fetal en plasma materno se sitúa por debajo del límite de detección de la técnica en el diagnóstico no invasivo, por lo que esta técnica no se aplica en el diagnóstico prenatal no invasivo.

### MLPA

La técnica MLPA (*multiplex ligation probe amplification*) [12] utiliza sondas (parejas de oligonucleótidos) que reconocen regiones específicas del gen a estudio, generalmente exones. El análisis de los datos se puede realizar mediante programas especializados (Coffalyser) que facilitan enormemente los cálculos y la interpretación.

Se emplea habitualmente en el diagnóstico prenatal de enfermedades monogénicas causadas por grandes deleciones/duplicaciones en un gen determinado. Un ejemplo es la distrofia muscular de Duchenne, en la que el 65–80% de las mutaciones causales son deleciones/duplicaciones que abarcan uno o más exones del gen DMD [13].

Debido a que el ADN fetal está fragmentado en el plasma materno, la técnica de MLPA no se emplea en el diagnóstico no invasivo.

### Arrays de hibridación genómica comparada (array CGH y CGH-array de SNPs)

La técnica de array CGH se basa en la hibridación equimolar de un ADN muestra (paciente) y un ADN control (referencia) del mismo sexo sobre un ADN molde que contiene múltiples secuencias localizadas a lo largo del genoma, asociadas con patología.

Permite la detección de variaciones en el número de copias (CNVs del inglés *copy number variations*) o grandes deleciones y duplicaciones a lo largo de todo el genoma o de regiones del genoma asociadas a patologías. La resolución del arrray CGH va a depender del tipo de array y del diseño. Ha demostrado ser muy eficiente en el diagnóstico de enfermedades monogénicas como la distrofia muscular de Duchenne [14] y discapacidad intelectual [15] en el diagnóstico prenatal invasivo. Esta técnica no se emplea en el diagnóstico no invasivo, ya que la muestra representa una mezcla de ADN fetal y materno.

Los CGH arrays de SNPs tienen una elevada sensibilidad. Permiten detectar inserciones y deleciones de menor tamaño y tienen como característica añadida la posibilidad de detectar pérdidas de heterocigosidad y detectar zonas del genoma asociadas a “*imprinting*”.

### Estudio de microsatélites mediante PCR

El análisis molecular de microsatélites se basa en la determinación del número de repeticiones de regiones específicas del genoma que son altamente polimórficas. Para ello, se lleva a cabo una amplificación mediante la reacción en cadena de la ADN polimerasa (PCR), utilizando cebadores marcados con fluorescencia de la región donde se localiza el microsatélite a estudio y mediante electroforesis capilar se analiza el tamaño de los fragmentos amplificados.

El estudio de microsatélites mediante PCR se realiza en los casos en los que no se conoce o no es posible el estudio de la mutación causal, por lo que se requiere un diagnóstico molecular indirecto de la enfermedad genética familiar, o en las enfermedades originadas por grandes expansiones de una región de repeticiones (trinucleótidos, la mayoría) como por ejemplo la distrofia miotónica tipo 1 o la ataxia de Friedreich.

En el caso de estas enfermedades, la técnica de PCR para el estudio de microsatélites se combina con la técnica *repeat primed PCR*, denominada *triplet repeat primed PCR* (TP-PCR) en el caso de estudiar la expansión de trinucleótidos (explicada más adelante).

Esta técnica no se puede realizar en diagnóstico no invasivo dado que el ADN fetal está fragmentado en el plasma materno.

### *Repeat primed* *PCR* *. Triplet repeat primed* *PCR* (TP-PCR)

Dado que la mayoría de las enfermedades originadas por la expansión de repeticiones se caracterizan por la expansión de trinucleótidos, nos referiremos a la técnica TP-PCR.

La técnica TP-PCR es una variante de la PCR con una elevada sensibilidad y especificidad para detectar la presencia o ausencia de expansión de trinucleótidos, gracias a que uno de los cebadores es complementario a la secuencia repetitiva de nucleótidos [16]. Los productos obtenidos mediante la TP-PCR se analizan mediante electroforesis capilar. En el caso de que exista expansión, se obtiene una imagen electroforética típica de escalera o cola de dragón, disminuyendo gradualmente la altura de los picos al aumentar el tamaño del número de repeticiones.

Esta técnica se emplea de manera complementaria a la PCR para el estudio de microsatélites en el diagnóstico prenatal invasivo de enfermedades causadas por expansión de un microsatélite como por ejemplo la distrofia miotónica tipo 1.

### Paneles de variantes patogénicas más prevalentes asociadas a una enfermedad concreta. Amplificación específica de alelos (ARMS, siglas en inglés: *amplification-refractory mutation system*)

En algunas patologías hay descritas una serie de variantes patogénicas que, en conjunto y teniendo en cuenta el ancestro poblacional, explican una determinada enfermedad en la mayoría de los individuos/familias afectados/as. Por ello, se han desarrollado reactivos comerciales que analizan en el mismo ensayo, las posiciones nucleotídicas en las que se localizan las alteraciones genéticas más comunes en patologías como la fibrosis quística, déficit de alfa-1-antitripsina o enfermedad de Stargardt. Esta estrategia diagnóstica se aplica en casos de sospecha de estas enfermedades en el feto, empleándose en el diagnóstico prenatal invasivo y utilizando como muestras el líquido amniótico o las vellosidades coriales. Una de las técnicas más utilizadas para estos ensayos es la amplificación específica de alelos (ARMS) que se basa en el uso de cebadores específicos en una PCR multiplex que detectan alelos normales o silvestres (*wild type*) y alelos mutados en el gen/genes estudiados [17].

Ante un hallazgo ecográfico que nos haga sospechar de una patología concreta se recomienda realizar, si es posible, el diagnóstico molecular (intestino hiperecogénico en la ecografía fetal/fibrosis quística [18]).

Estos paneles analizan únicamente las variantes patogénicas más comunes asociadas a la enfermedad, no el gen o genes completos, por lo que no nos permiten descartar la patología. Por esto, es importante establecer e indicar en el informe la sensibilidad de la técnica en nuestra población a estudio y/o el riesgo residual de portador, al igual que todas las técnicas que no descartan una patologia concreta.

### PCR a tiempo real con sondas Taqman

La sonda Taqman es un oligonucleótido complementario a una región diana, que lleva unido un grupo emisor de fluorescencia a su extremo 5′ y un grupo supresor de fluorescencia a su extremo 3′ y se emplean cebadores que delimitan la región de unión de la sonda. Durante la amplificación la sonda hibridada se escinde, emitiéndose fluorescencia al alejarse el fluoróforo de la molécula supresora, que está en relación directa con la aparición de producto amplificado específico [19].

Es una técnica que ha sido ampliamente utilizada en el diagnóstico prenatal, requiriendo un diseño específico para cada variante patogénica y/o diagnóstico. Sin embargo, recientemente ha sido desplazada por la PCR digital en muchas situaciones.

En el diagnóstico prenatal no invasivo se necesitan un número mayor de ciclos de amplificación en el análisis mediante PCR a tiempo real para obtener una cantidad suficiente del ADN fetal, minoritario en la muestra de la embarazada. Se emplea en este contexto para la detección de genes ausentes en la madre, como en el caso de la determinación del sexo fetal o la determinación de la existencia del gen *RHD* en el feto.

Se considera un método con sensibilidad y especificidad suficiente para dar resultado negativo ante la no detección del cromosoma Y y/o el gen *RHD* en el feto, siempre que se confirme en una segunda muestra en los casos de ausencia de ambos genes. A partir de la semana 10 de gestación se considera que existe suficiente ADN fetal para este diagnóstico [20].

### PCR digital

La PCR digital está basada en la distribución del ADN molde objeto de análisis en un número elevado de particiones dando lugar a reacciones individuales de PCR [21, 22]. Se realizan entre 10.000 y 20.000 PCRs individuales.

Presenta una alta sensibilidad, detectando incluso variantes existentes al 0.1% [23], lo que permite su aplicación en el diagnóstico o test prenatal no invasivo [10] y una cuantificación directa de la diana objeto de estudio.

La PCR digital permite detectar variantes causales previamente conocidas heredadas de la madre, de origen paterno o *de novo* con riesgo de recurrencia.

El análisis de secuencias exclusivas paternas mediante PCR digital requiere un enfoque de detección, mientras que el estudio de las regiones genómicas maternas requiere calcular la dosis de la fracción alélica fetal en el plasma materno (RMD, del inglés *relative mutation dosage*) que determina si existe o no un desequilibrio en la fracción alélica fetal y así establecer el genotipo del feto [10].

Esta técnica ha sido validada demostrando una alta sensibilidad y una exactitud del 100% para detectar los alelos de origen paterno y de un 96% al hacer el análisis de RMD para detectar los alelos de origen materno, tanto en enfermedades autosómicas como ligadas al cromosoma X [23].

### HRM (*high resolution melting*) y COLD-HRM

La técnica de HRM (de sus siglas en inglés *high resolution melting*) se basa en la realización de una PCR a tiempo real en presencia de agentes intercalantes del ADN de doble hélice, que únicamente emiten fluorescencia cuando se intercalan en la doble hebra y dejan de emitirla al disociarse el ADN en cadenas sencillas [24]. Nos permite diferenciar las muestras de ADN acorde a la curva “*melting*” y Temperatura de *melting* (TM), proporcionando alta sensibilidad y resolución a los perfiles obtenidos con la técnica HRM. Una vez comprobado por secuenciación el resultado del producto amplificado, la técnica HRM se considera igual de sensible que el “*gold estándar*” para las mismas condiciones y para la mutación concreta estudiada.

La técnica COLD-HRM es una variante del análisis HRM que emplea en una segunda fase de PCR la temperatura “*melting*” como temperatura de desnaturalización. Se utiliza para detectar las variantes genéticas que están en baja proporción en la muestra. Se llega a detectar ADN mutado que se encuentra incluso al 2% respecto al silvestre, por lo que es una técnica apta para diagnóstico no invasivo de variantes puntuales fetales ausentes en la madre [25, 26].

### Secuenciación masiva (NGS)

La NGS (por sus siglas en inglés, *next generation sequencing*) o secuenciación masiva es una tecnología de alto rendimiento que permite el análisis o lectura de nucleótidos de millones de fragmentos de ADN al mismo tiempo y de manera rápida. Permite detectar variantes puntuales, pequeñas deleciones e inserciones y, mediante análisis especiales, permite detectar variaciones en el número de copias (CNVs) [27]. Actualmente se recomienda confirmar las CNVs detectadas en NGS mediante MLPA o array.

La secuenciación masiva es una técnica que se emplea tanto en el diagnóstico prenatal invasivo como no invasivo, con enfoques totalmente diferentes.

#### NGS en el diagnóstico prenatal invasivo

Las aproximaciones diagnósticas utilizadas en diagnóstico prenatal son:

– Secuenciación de paneles: permite el análisis de genes asociados a determinadas patologías.
– Secuenciación del exoma clínico: secuencia las regiones codificantes de los genes descritos previamente asociados con patología.
– Secuenciación del exoma completo: secuencia las regiones codificantes de todos los genes del genoma humano.

Actualmente se recomienda el uso de la secuenciación masiva en el diagnóstico prenatal invasivo de enfermedades monogénicas en la rutina clínica, sólo en determinadas circunstancias [28], [29], [30]. El American College of Medical Genetics and Genomics (ACMG) recomienda valorar la secuenciación de exoma cuando no se ha conseguido el diagnóstico con estudios genéticos rutinarios (cariotipo o array de ADN genómico) en un feto con múltiples anomalías que sugieran origen genético [31]. Los datos publicados en diagnóstico prenatal mediante secuenciación de exoma se limitan a pequeñas series de casos. Drury y colaboradores identifican anomalía genética hasta en un 20–30% de los fetos con múltiples anomalías con resultados normales en los estudios genéticos convencionales [32].

Por otro lado, la secuenciación masiva ha permitido de manera rápida la secuenciación de los genes implicados en una determinada patología en fetos en los que se ha reconocido un determinado fenotipo [33].

Varios estudios de exoma en diagnóstico prenatal sostienen que la secuenciación en tríos (secuenciación de la muestra del feto y de los padres biológicos de manera simultánea) mejoran el rendimiento diagnóstico y la rapidez de análisis [32, 34].

#### NGS en el diagnóstico prenatal no invasivo

Está descrito que los dos factores principales que afectan a la precisión del estudio son: la fracción fetal que debe sobrepasar un umbral (recomendado el 4%) y la profundidad de secuenciación del cfDNA pues para cuantificar fracciones pequeñas dentro del ADN circulante materno es necesario una profundidad mucho mayor que para un estudio genético cualitativo.

##### Paneles de NGS-dosis relativa de haplotipo (RHDO, del inglés *relative haplotype dosage analysis*)

Los paneles de NGS se emplean en el estudio de variantes heredadas de la madre en trastornos de un sólo gen mediante el método de RHDO [10]. Mientras que RMD (RMD, del inglés *relative mutation dosage*) cuantifica directamente las relaciones alélicas para una variante específica, el análisis RHDO se usa para calcular las relaciones alélicas en el plasma materno para bloques de haplotipos, de modo que, en lugar de buscar la variante específica, se realiza el análisis para el haplotipo vinculado a la variante causal.

Para el análisis RHDO es necesario el estudio de los haplotipos paternos y maternos, así como determinar el haplotipo vinculado a la variante patogénica (probando afecto), para después deducir los haplotipos que ha heredado el feto. Cuanto mayor es el número de los SNPs incluidos en el análisis, mayor es el poder estadístico. Cuando el porcentaje de ADN fetal en el plasma es superior al 4% se ha descrito que se consiguen sensibilidades del 100% [35].

El RHDO permite un enfoque altamente efectivo para detectar variantes genéticas complejas (genes con pseudogenes, grandes deleciones genéticas y variantes patogénicas localizadas dentro de elementos repetitivos), en los que resulte imposible el análisis directo de la variante.

El diagnóstico prenatal no invasivo mediante el análisis RHDO se aplica en patologías como la distrofia muscular de Duchenne y Becker [36], fibrosis quística o la atrofia muscular espinal [37].

##### Secuenciación del exoma/genoma completo en diagnóstico prenatal no invasivo

La secuenciación del exoma o genoma en el diagnóstico prenatal no invasivo de enfermedades monogénicas aún no está implantada en la rutina clínica, no obstante, se están haciendo estudios que cada vez consiguen una mayor eficiencia [38].

**Figura 1:**
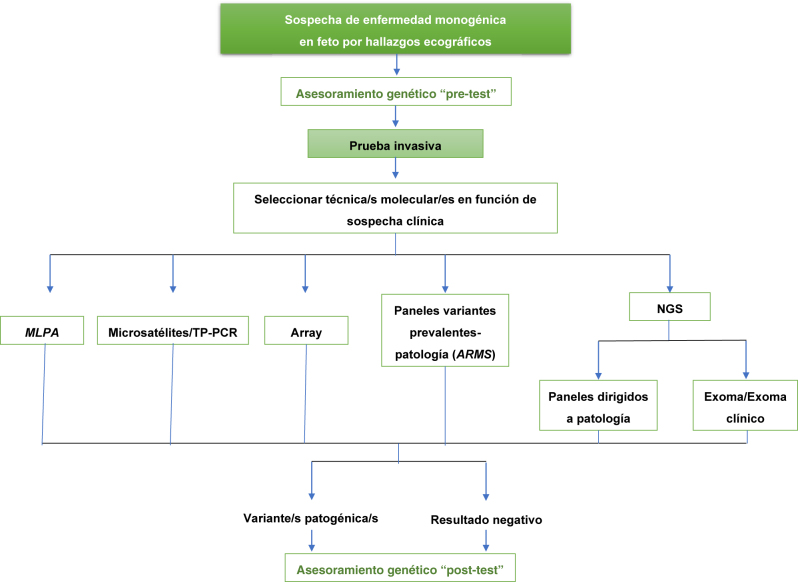
Algoritmo diagnóstico ante la sospecha de una enfermedad monogénica en feto por hallazgos ecográficos. ARMS, *amplification-refractory mutation system*; MLPA, *multiplex ligation probe amplification*; NGS, *next generation sequencing*; TP-PCR, *triplet repeat primed PCR*.

En las Figuras 1 y 2 proponemos los algoritmos diagnósticos en función de la indicación del estudio genético.

**Figura 2:**
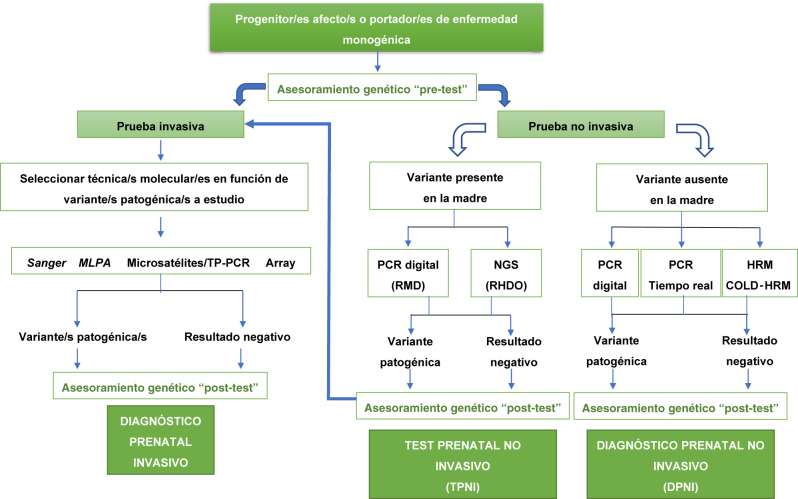
Algoritmo diagnóstico para descartar una enfermedad monogénica en feto en el que uno de los progenitores o ambos son afecto o portadores de una enfermedad monogénica. ARMS, *amplification-refractory mutation system*; HRM, *high resolution melting*; MLPA, *multiplex ligation probe amplification*; NGS, *next generation sequencing*; NIPD, *non invasive prenatal diagnosis*; NIPT, *non invasive prenatal test*; RHDO, *relative haplotype dosage analysis*; RMD, *relative mutation dosage*; TP-PCR, *triplet repeat primed PCR*.

## Limitaciones técnicas

### Limitaciones de las técnicas empleadas en el diagnóstico prenatal invasivo

– Las diferentes técnicas van a ser capaces de detectar determinados tipos de variantes (TP-PCR: expansión de repeticiones; MLPA: grandes deleciones y duplicaciones, etc.) e incapaces de detectar otras, por lo que van a presentar esa limitación por sí misma.
– No detectan mosaicismos de bajo grado o compensados, ni contaminación celular materna (MLPA y aCGH).
– El análisis de microsatélites y TP-PCR para el estudio de enfermedades causadas por expansión de repeticiones, sólo permite detectar presencia o ausencia del alelo patológico y no es capaz de cuantificar el número de repeticiones de grandes expansiones [16].
– Los paneles de variantes patogénicas comunes o prevalentes de una patología nos permiten analizar una serie de variantes determinados. Para ampliar el estudio y aumentar la sensibilidad del análisis necesitaremos de pruebas complementarias como la secuenciación Sanger, la NGS o el MLPA,
– La interpretación de las variantes identificadas es compleja y en muchas ocasiones se obtienen variantes de significado incierto que pueden crear ansiedad en la familia y dificultar la toma de decisiones.
– Las limitaciones de la técnica de NGS:
  – Posibilidad de obtener baja cobertura en genes claves, con posibles resultados falsos negativos.
  – Detección de hallazgos incidentales no relacionados con el fenotipo [39].
  – No es capaz de detectar determinadas variantes patogénicas (grandes reordenamientos genéticos, variantes en regiones no incorporadas en la captura).
– El diagnóstico prenatal requiere un resultado con un tiempo de respuesta de días o semanas, por lo que la aplicación de determinadas técnicas moleculares va a estar limitada a determinadas situaciones.

### Limitaciones de las técnicas de NIPD/NIPT

Estas técnicas tienen la peculiaridad que al hacerse en el plasma materno, la proporción de ADN fetal será la mayor limitante en base a las sensibilidades de las técnicas empleadas.

– Para el diagnóstico de una variante inexistente en la madre es necesario un 2% de concentración de ADN fetal en el plasma materno para PCR a tiempo real y un 0,2% si se emplea la PCR digital [23, 40].
– Para el diagnóstico de una variante inexistente en la madre con fluoróforos intercalantes del ADN (diagnosticados por curva *melting*), la sensibilidad es del 12% para la HRM convencional y del 2% para la COLD-HRM [25, 26]
– Cuando el abordaje de sospecha diagnóstica es cuantitativo se suele requerir al menos un 4% de ADN fetal circulante [5] en la gestante.
– Hay que considerar los factores que pueden hacer disminuir el porcentaje de ADN fetal en la gestante, como la obesidad materna o la existencia de algún proceso inflamatorio en la madre que aumente temporalmente el ADN circulante materno enmascarando el ADN fetal, la existencia de un proceso neoplásico maligno o un proceso autoinmune activo en la madre [6], [7], [8]. Pero no sólo estos ya que hay un importante número de factores que afectan a la fracción fetal: determinadas características maternas, características placenta-feto, factores experimentales y métodos de cálculo [9].
– La técnica de NGS en NIPD/NIPT:
  – Es bastante laboriosa y costosa ya que necesita una profundidad de lecturas en la secuenciación suficiente para detectar el genotipo de ADN fetal.
  – En la técnica de RHDO se necesita un probando (familiar afecto) del que no siempre se dispone, para vincular el diagnóstico.

Todas las limitaciones inherentes a la/s técnica/s empleadas para el diagnóstico prenatal, deben ser detalladas exhaustivamente durante la fase del asesoramiento genético pre-prueba.

## Requisitos y recomendaciones analíticas

Los laboratorios que realicen diagnóstico prenatal deben tener amplia experiencia en el diagnóstico de la patología en concreto: utilizando métodos validados y participando anualmente en controles de calidad externos.

### Recomendaciones en el diagnóstico prenatal invasivo

– Se debe realizar el diagnóstico por duplicado en el feto y, si es posible, por dos métodos diferentes. Se recomienda dividir la muestra de líquido amniótico o vellosidad corial en dos alícuotas a la llegada al laboratorio y procesarlas de manera independiente.
– Descartar toda contaminación materna en la muestra fetal.
– El caso índice y los padres se deben procesar en paralelo con la muestra fetal, siempre que sea posible.
– Se debe emitir el resultado en un plazo de una semana desde que se recibe la muestra en el laboratorio, para la mayoría de los estudios.
– El informe debe ser conciso y claro y debe especificar [41]:
  – Que se ha descartado la presencia de contaminación materna.
  – La repercusión clínica del resultado molecular.
  – Siempre que se realice un diagnóstico indirecto, es importante reflejar en el informe el riesgo.

### Recomendaciones en el diagnóstico prenatal no invasivo

La determinación de alteraciones genéticas en el ADN circulante requiere unas condiciones preanalíticas muy estrictas.

– Se recomienda utilizar plasma (extraído en EDTA) si se va a realizar secuenciación masiva del ADN circulante en sangre. Para el diagnóstico de presencia de genes ausentes en el ADN materno, como es el caso del RHD y el sexo fetal, se puede utilizar plasma (extraído en EDTA) o suero y para PCR digital y HRM se usa suero, aunque el plasma también valdría [42].
– Es importante que el tubo llegue al laboratorio dentro de las primeras cuatro a seis horas tras la extracción; a menos que se usen tubos que protejan la integridad celular [42].
– Se aconseja conservar el plasma a −80 °C y el ADN circulante ya extraído a −20 °C, evitando repetir ciclos de congelación y descongelación [42].
– Es recomendable obtener fragmentos de ADN de 100 a 400 pb de longitud, que seleccionan preferentemente el ADN fetal (existen kits específicos de extracción de ADN circulante) [42].
– La fracción de ADN fetal debe ser superior al 4% [5] si se va a realizar secuenciación masiva para cuantificación, si no se recomienda un diagnóstico fetal con técnica invasiva. Cuando se usan técnicas de PCR a tiempo real, al tratarse de un diagnóstico cualitativo, la fracción fetal puede ser menor, siendo suficiente un 2% si se hace COLD-HRM [25, 26] y tan sólo el 0.2% si se hace PCR digital [23].
– Es aconsejable, en los estudios de NGS, para evitar falsos negativos debido a concentraciones muy bajas de ADN circulante fetal, identificar los alelos heredados por vía paterna, o bien demostrar la presencia de ADN fetal y cuantificar su porcentaje.
– Si hay placenta que provoca preeclampsia es necesaria una técnica invasiva para un diagnóstico fetal.
– No se puede descartar la existencia de mosaicismos confinados a la placenta ya que el componente fetal proviene de la placenta, que pueden dar lugar a posibles falsos positivos o resultados no concluyentes.
– Para evitar falsos positivos, debido a la posibilidad de la existencia de un segundo embrión previo (no detectado) conocido como “gemelo evanescente o desaparecido”, que puede haber sido afecto, se debe excluir la presencia de un saco gestacional vacío mediante una exploración ecográfica.
– Descartar posibles fuentes de ADN foráneo en la gestante, como es el caso de mujer receptora de un trasplante de órgano.
– El NIPD no es adecuado para su uso en embarazos múltiples, excepto en casos donde hay hallazgos discordantes de ultrasonido [5].

Las técnicas empleadas en el diagnóstico prenatal son muy diversas y cada vez más complejas, planteando nuevos retos técnicos, analíticos, legales y éticos.

## Asesoramiento genético

Las definiciones y detalles del asesoramiento genético prenatal se recogen en la ley de investigación biomédica [43], en las guías de la *American Society of Human Genetics* (https://www.acmg.net/ACMG/Medical-Genetics-Practice-Resources/Practice-Guidelines.aspx) y en el convenio europeo sobre derechos humanos y biomedicina [44], entre otros (https://www.nsgc.org/page/specialty-areas).

El cometido de la consulta de asesoramiento genético prenatal es informar acerca de cualquier defecto congénito que pueda haber en futuros nacidos: anomalía morfológica, estructural, funcional o molecular que pueda estar presente al nacer (pudiendo manifestarse más tarde), evidente en el aspecto físico externo y/u órganos internos, familiar o esporádica, hereditario o no, única o múltiple. Las consultas de asesoramiento genético prenatal se solicitan ante la sospecha de una alteración genética en el feto o la posibilidad de transmisión de una alteración genética a los hijos y pueden referirse por tanto al periodo preconceptivo, preimplantacional y/o gestacional de un individuo. El objetivo es, principalmente, el diagnóstico o predicción de la existencia de una enfermedad genética en el feto, muy frecuentemente asociada a discapacidades graves. Debe existir evidencia científica suficiente para considerar la alteración genética patogénica y responsable de la enfermedad.

El asesor genético debe cerciorarse de que la información ha sido comprendida por las personas asesoradas, que puedan tomar una decisión libre e informada y en ningún caso tenga una naturaleza directiva. El asesoramiento genético requiere un imprescindible cumplimiento de la legislación y normativa vigentes [2, 43], [44], [45].

Otros matices de las consultas de asesoramiento genético prenatal son la premura que exige la emisión de un informe lo más claro posible, realizar una interpretación absolutamente estricta y con las evidencias científicas más actuales en la fecha de la consulta. En el período prenatal la imagen ecográfica proporciona los únicos datos fenotípicos disponibles del feto que suelen ser escasos y poco definidos pero fundamentales para enfocar e interpretar los resultados del diagnóstico genético.

Son imprescindibles al menos una consulta antes de llevar a cabo cualquier prueba genética (asesoramiento “pre-test”), y otra cita posterior para entregar los resultados obtenidos, explicando lo que de ellos se deriva (asesoramiento “post-test”).

Actualmente se recomienda que los laboratorios de Genética sigan las normas de acreditación y certificación ISO 17025, ISO 15189 e ISO 9001, existiendo además otras guías y recomendaciones como las que emite la Asociación Española de Genética Humana o la de OECD [46], [47], [48], [49].

### Consulta de asesoramiento previa a la prueba genética

En la consulta de asesoramiento genético previa a la prueba hay una serie de puntos clave:

1. Exposición del motivo de solicitud.
2. Recopilación de datos personales, familiares, informes clínicos, analíticos y genéticos que el solicitante/familia tuviera, en relación con el motivo de su consulta. La construcción de un árbol genealógico lo más completo posible es esencial.
3. Exposición de la estrategia diagnóstica, detallando el objetivo del estudio, su rentabilidad diagnóstica y el tipo de muestra, los beneficios de llevar a cabo la prueba genética y la posibilidad de confirmar el diagnóstico, pronóstico y tratamiento. Es esencial describir la precisión y limitaciones de la prueba indicada y enmarcar la prueba entre otras pruebas alternativas. Debemos explicar los posibles resultados, si a partir de las pruebas podemos identificar o no el agente causal único de la enfermedad, obtener resultados de significado incierto, acerca de los cuales no hay información previa para poder asegurar sus consecuencias [50] y detectar hallazgos inesperados con asociación demostrada a la aparición de otras enfermedades que no tengan relación con la sospecha diagnóstica por la que acudieron a consulta. Se deben explicar las posibles implicaciones que puedan derivar de los resultados de la prueba a otros miembros de la familia.
4. Obtención del consentimiento informado. La gestante declara que ha entendido toda la información recibida y su participación es voluntaria, da su consentimiento para la realización de la prueba propuesta, expresa su decisión acerca de la conservación de la/s muestra/s y si desea ser informada de hallazgos incidentales y/o de resultados inciertos.

Se ha consensuado el recoger el consentimiento informado por escrito y conservarlo en el historial de la paciente. El asesor debe saber que constituye una obligación legal y moral con los individuos atendidos en este tipo de consultas [51].

### Consulta de asesoramiento posterior a la prueba genética

En la consulta de asesoramiento posterior a la prueba se trasmitirá a la gestante y/o pareja la información generada en los estudios genéticos realizados. Debe hacerse de forma clara y comprensible, exponiendo las consecuencias para el nacido y su familia, riesgo de recurrencia en la descendencia, así como posibilidades de prevención primaria o secundaria, tanto en el periodo prenatal como postnatal.

Además de la explicación de los resultados genéticos obtenidos, debe integrarse la información analítica y clínica del feto relativa al proceso llevado a cabo. Es frecuente que surja la necesidad y utilidad de ofrecer a la familia estudios de segregación de las variantes genéticas identificadas en los diferentes miembros, afectos y no afectos.

Es una labor esencial de las consultas de asesoramiento la coordinación de la atención o seguimiento clínico por tantos especialistas como requieran el feto, el posible nacido, sus progenitores incluso otros familiares, para el adecuado manejo de cada situación.

La justificación de un diagnóstico genético lo más temprano posible es creciente, ante la aparición de prácticas terapéuticas que puedan incluso permitir la corrección total de la patología o al menos la moderación de alguna de sus manifestaciones.

## Conclusiones

El diagnóstico genético prenatal de las enfermedades monogénicas se ha realizado tradicionalmente de manera invasiva pero es una realidad que se puede realizar a partir de una muestra de la sangre periférica de la gestante, sin afectar físicamente a los tejidos de origen fetal. Evitando el estrés de la gestante, así como el riesgo asociado de pérdida fetal, actualmente aceptado desde 0.11% postamniocentesis y del 0.22% postbiopsia corial [52] a 0.35% tanto post-amniocentesis como post-vellosidad corial [53] dependiendo los estudios. El abordaje molecular del diagnóstico prenatal debe ser el adecuado para cada caso, por lo que deberemos conocer las técnicas disponibles y sus limitaciones para ofrecer un diagnóstico, con el enfoque idóneo. Gracias a la evolución de las tecnologías de secuenciación masiva y PCR digital cada vez es más asequible el diagnóstico genético de enfermedades monogénicas. Requiriendo una constante actualización homogénea de los laboratorios.

Todo diagnóstico genético prenatal debe ir acompañado de un asesoramiento genético, ofrecido por un profesional cualificado, que permita a la gestante tomar decisiones y comprender la repercusión de los resultados genéticos obtenidos.
